# Like Alice in Wonderland, ROOT-ExM enlarges root tips for a closer look

**DOI:** 10.1093/plcell/koaf078

**Published:** 2025-04-02

**Authors:** Laura Arribas-Hernández

**Affiliations:** Assistant Features Editor, The Plant Cell, American Society of Plant Biologists; Instituto de Hortofruticultura Subtropical y Mediterránea La Mayora (IHSM), Universidad de Málaga ‐ Consejo Superior de Investigaciones Científicas (UMA‐CSIC), Boulevard Louis Pasteur 49, 29010 Málaga, Spain

The caterpillar said to Alice: “One side will make you grow taller, and the other side will make you grow shorter” (Carroll 1865). This could also be said of a pair of binoculars or a microscope, except that the magnification given by these instruments is optical and, unlike Alice, the specimen remains the same size… at least until now. In new work, Magali S. Grison and colleagues (Grison et al. 2025) have been able to physically enlarge Arabidopsis root tips to see subcellular details with super-resolution using a conventional microscope.

Super-resolution microscopes can overcome the resolution limit of conventional optical microscopy imposed by the physics of light diffraction. Thanks to this property, fluorescently labeled macromolecules can be visualized with unprecedented detail. However, the cost and the expertise needed for using such instruments impose another limitation for many researchers. An alternative is expansion microscopy (ExM) (Chen et al. 2015), a sample preparation technique that can circumvent the diffraction limit by physically increasing the sample size rather than enhancing the power of the microscope. The trick to make the specimen grow involves introducing a water-absorbent polymer into the fixed tissue and treating the sample with Proteinase K to allow expansion. As the gel swells, the distance between fluorophores within the sample increases, thereby improving the resolution by a factor that is proportional to the expansion, which ranges from 4- to ∼20-fold.

Since its introduction in 2015 (Chen et al. 2015), ExM has been used to image proteins, RNA, and lipids in different animal tissues and yeast. Plants, on the other hand, have been left behind because of the properties of the plant cell wall: a rigid matrix that is hard to penetrate and resistant to expansion. In a Breakthrough Report, Magali S. Grison and colleagues (Grison et al. 2025) have overcome this obstacle, successfully expanding Arabidopsis root tips through a method named ROOT-ExM. The robustness of this technology is further supported by a companion article in this issue by Gallei et al. (2025), who independently report a similar strategy named PlantEx (highlighted by Chakraborty 2025).

ROOT-ExM combines an ExM variant that preserves genetically encoded fluorescent proteins with a whole-mount immunolocalization protocol for Arabidopsis roots that partially digests the cell wall to facilitate antibody penetration and tissue expansion. Altogether, ROOT-ExM involves 7 main steps (Fig. 1): 1) chemical fixation of seedlings; 2) dissection of root tips; 3) mild digestion of cell walls in optimized conditions to preserve the integrity of the tissue; 4) labeling the molecules of interest; 5) overnight acrylamide/bisacrylamide incubation to enhance monomer penetration across the tissue, followed by standard gelation with a polymerizing agent; 6) proteinase K digestion to allow expansion; and 7) tissue expansion. Following this protocol, the authors achieved a ∼4-fold expansion of the tissue, a ratio that remained fairly constant across the entire root meristem with minimal tissue distortion (Grison et al. 2025).

**Figure.**
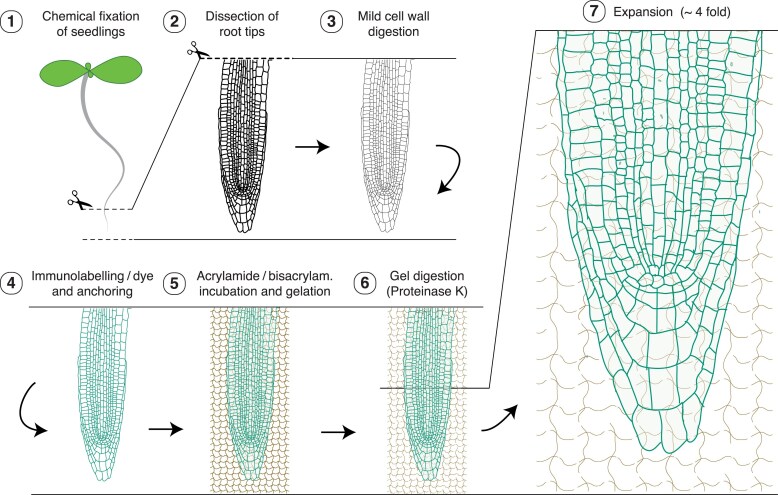
Key steps of ROOT-ExM. Schematic representation of the 7 main steps in the ROOT-ExM sample preparation protocol. Figure credit: L. Arribas-Hernández, inspired by the representation in Grison et al. (2025), using the root tip graph from Erin Sparks, available on Figshare (https://figshare.com/articles/figure/Arabidopsis_root_anatomy/4688344) under a CC BY 4.0 license.

The article by Grison et al. (2025) shows a collection of beautifully resolved subcellular structures, for example, nuclei, microtubule networks during cell division, membranous compartments such as the endoplasmic reticulum, Golgi cisterna and cell plate formed during cytokinesis, and cell wall–encased structures like plasmodesmata. To visualize these structures, the authors used 3 different labeling techniques: 1) intrinsic tissue fluorescence from YFP-tagged proteins; 2) dyes such as DAPI or calcofluor white; and 3) immunofluorescence with antibodies, for example, against alpha-tubulin (microtubules), KNOLLE (cell plate vesicles), SUN (nuclei), or callose (plasmodesmata). In all cases, the subcellular compartments exhibited good structural preservation compared with non-expanded samples, and multiple color labeling was also achieved. Importantly, the measurement of key features such as cell wall thickness or plasmodesmata diameter agreed with the size estimated by electron microscopy or super-resolution microscopy based on stimulated emission depletion (STED) (Vicidomini et al. 2018) in previous works. In fact, a direct comparison between ROOT-ExM and STED microscopy showed that both techniques achieve similar resolution for small structures (Grison et al. 2025).

ROOT-ExM is compatible with global protein fluorescent labeling (panlabelling) using N-hydroxysuccinimide ester-dye conjugates. In particular, the STED-compatible N-hydroxysuccinimide ester-ATTO647 allowed Grison et al. to visualize ultrastructural details by 2-dimensional STED of ROOT-ExM–expanded samples. The combination of the 2 techniques spatially resolved details such as mitochondria cristae or bridge-like structures between cells that were confirmed to be plasmodesmata (Grison et al. 2025). In addition to STED, ROOT-ExM can be combined with lattice light sheet microscopy. This technique provides enhanced resolution in the z-axis, which is otherwise resolved with less detail than the x/y plane by conventional microscopes. As a case study for this technique combination, the authors chose the cell plate, a membrane-polysaccharide compartment that expands radially during cytokinesis to eventually connect to the parental side walls and complete cell division. As expected, lattice light sheet microscopy of ROOT-ExM–expanded samples revealed the intricate details of the tubulo-vesicular fenestrae membrane structure, as well as cargo vesicles—of sizes below the light diffraction limit—as they approach the plate (Grison et al. 2025).

Despite the spectacular images displayed by Grison et al. in their article (2025), the technique still has limitations. It is unlikely that plant tissues with lignified secondary walls can be expanded, and expansion may be uneven in organs composed of tissues with very different mechanical properties. Furthermore, the aldehyde-based tissue fixation step (Figure) may introduce artifacts that could be avoided by cryofixation, an improvement to the technique that may become feasible soon. Nevertheless, ROOT-ExM's ability to achieve nanoscale resolution using conventional microscopes—without the need for specialized equipment—and its compatibility with other microscopy techniques represent clear advantages for many researchers.

## Data Availability

No new data were generated or analysed in support of this research.

## Dive Curated Terms

The following phenotypic, genotypic, and functional terms are of significance to the work described in this paper:

DAPI CHEBI: CHEBI:51231

ExM Gramene: expansion microscopy
ExM Araport: expansion microscopy

ROOT-ExM Gramene: root expansion microscopy
ROOT-ExM Araport: root expansion microscopy
